# Sensitive, Real-time and Non-Intrusive Detection of Concentration and Growth of Pathogenic Bacteria using Microfluidic-Microwave Ring Resonator Biosensor

**DOI:** 10.1038/s41598-018-34001-w

**Published:** 2018-10-25

**Authors:** Rakesh Narang, Sevda Mohammadi, Mehdi Mohammadi Ashani, Hamid Sadabadi, Hossein Hejazi, Mohammad Hossein Zarifi, Amir Sanati-Nezhad

**Affiliations:** 1 BioMEMS and Bioinspired Microfluidic Laboratory, Department of Mechanical and Manufacturing Engineering, University of Calgary, Calgary, AB T2N 2N1 Canada; 2 Microelectronics and Advanced Sensors Laboratory, School of Engineering, University of British Columbia, Kelowna, BC V1V 1V7 Canada; 3 Biomedical Engineering Graduate Program, University of Calgary, 2500 University Dr. NW, Calgary, AB T2N 1N4 Canada; 4 Center for BioEngineering Research and Education, University of Calgary, Calgary, AB T2N 1N4 Canada; 5 Wireless Fluidics Inc, Edmonton, AB Canada; 6 Subsurface Fluidics and Porous Media Laboratory, Chemical and Petroleum Engineering, University of Calgary, Calgary, AB T2N 1N4 Canada

## Abstract

Infection diagnosis and antibiotic susceptibility testing (AST) are time-consuming and often laborious clinical practices. This paper presents a microwave-microfluidic biosensor for rapid, contactless and non-invasive device for testing the concentration and growth of *Escherichia Coli* (*E. Coli*) in medium solutions of different pH to increase the efficacy of clinical microbiology practices. The thin layer interface between the microfluidic channel and the microwave resonator significantly enhanced the detection sensitivity. The microfluidic chip, fabricated using standard soft lithography, was injected with bacterial samples and incorporated with a microwave microstrip ring resonator sensor with an operation frequency of 2.5 GHz and initial quality factor of 83 for detecting the concentration and growth of bacteria. The resonator had a coupling gap area on of 1.5 × 1.5 mm^2^ as of its sensitive region. The presence of different concentrations of bacteria in different pH solutions were detected via screening the changes in resonant amplitude and frequency responses of the microwave system. The sensor device demonstrated near immediate response to changes in the concentration of bacteria and maximum sensitivity of 3.4 MHz compared to a logarithm value of bacteria concentration. The minimum prepared optical transparency of bacteria was tested at an OD_600_ value of 0.003. The sensor’s resonant frequency and amplitude parameters were utilized to monitor bacteria growth during a 500-minute time frame, which demonstrated a stable response with respect to detecting the bacterial proliferation. A highly linear response was demonstrated for detecting bacteria concentration at various pH values. The growth of bacteria analyzed over the resonator showed an exponential growth curve with respect to time and concurred with the lag-log-stationary-death model of cell growth. This biosensor is one step forward to automate the complex AST workflow of clinical microbiology laboratories for rapid and automated detection of bacteria as well as screening the bacteria proliferation in response to antibiotics.

## Introduction

Bacterial infection is a common problem throughout hospitals around the worlds^1^. Every hour of delay in antibiotic treatment increases the mortality rate of patients by 7.6% with sepsis and septic shocks^2^. The failure to diagnose early and critical stages of bacterial infections can be detrimental to a patient’s health and potentially fatal. The existing methods of diagnosing infections and performing antibiotic susceptibility testing (AST) suffer from a time-consuming and laborious process, typically taking up to 2–5 days to obtain accurate and reliable results^3–5^. Patients typically start showing symptoms of sepsis when blood concentration of bacteria reaches between 1–100 CFU/mL^6^. Several detection methods have been developed for bacteria detection and AST, including optical imaging^7,8^, cell counting^9–13^, Coulter counters^14^, pH monitoring^15^, magnetic^16^, fluorescent^17–19^, electrochemical^20^, or bioluminescence^17^ detection, which all require extensive image or signal processing, and are almost undesirable for clinical applications. Furthermore, their instrumentation can be large, expensive, laborious to work with and lack the potential for miniaturization and subsequent development for point-of-care applications. The lack of standardization of protocols also create discrepancies in results. There are still crucial needs to develop diagnostic devices compatible with the workflow of clinical microbiology laboratories with the capability of rapid, real-time, high-throughput and contactless detecting the concentration and growth of bacteria.

The advent of lab-on-a-chip microfluidic technology has so far revolutionized clinical analysis, medical research and diagnostics fields. Due to several inherent characteristics of microfluidic technologies such as portability, less patient sample requirements, miniaturization, minimizing user intervention, cost-effectiveness, enhanced sensitivity and specificity, and higher throughput, they have been recently used as a potent platform for multiple medical applications including clinical microbiology. Clinical analysis of blood and urine bioassays for monitoring infectious diseases, and drug discovery and development, microbiology and pathology studies are becoming fully automated with the increased efficiency of clinical practices^21–29^. Dedicated work has now gone into the development of microfluidic chips with the ability to detect bacteria^30^ and bacterial growth^8^ with high sensitivity, and perform rapid and accurate AST through employing accurate gradients of antibiotics^31,32^. Furthermore, through multiplexing, a multitude of assay combinations are possible in the realm of microfluidic-based biosensing^33^. Contemporary microfluidic devices are being combined with electromagnetic technologies to develop highly sensitive electromagnetic sensors for the detection of cells and molecules.

Microwave-based resonator devices have recently demonstrated a significant potential for biosensing^26,33–36^. They can translate a variation in dielectric properties of adjacent materials into quantifiable electrical signals such as resonant frequency and resonant amplitude in a remote non-contact manner. The present resonant-based bacteria sensing devices operate mostly in optical, microwave, and terahertz spans of electromagnetic spectrum among which, planar microwave resonators have grabbed extensive attention in recent years^33,36,37^. Through their intrinsic advantages, including simple and low-cost fabrication process, compatibility with other state-of-the-art technologies such as printed circuit boards, complementary metal oxides semiconductors (CMOS), and microfluidic lab-on-a-chip systems^38^, their merits increase significantly as biosensors in biomedical applications. Several groups including ours have integrated planar microwave sensing structures with microfluidic technology to demonstrate outstanding potential for sensing and monitoring^36,37,39^, where the resonator is able to detect changes in the electric field as a result of changes in liquid inside the microfluidic channel. Nicolic-Jarik *et al*.^40^ demonstrated a transmission line resonator alongside with radiofrequency (RF) readout/setup circuitry to detect small capacitive changes of 650 zF in an interdigitated capacitor structure within microfluidics channel. Nakouti *et al*.^41^ also employed interdigitated capacitor structure embedded within microfluidic channels to detect bacterial contaminations in water based on reflection pattern of microwave power. Utilizing a planar microwave resonator in conjunction with microfluidics as a biosensor to assist in infection diagnosis and AST analysis could prove to increase clinical efficiency and decrease infectious fatalities. Further studies are needed to prove that this is a viable pathway for the future of AST biosensors.

**Figure 1:**
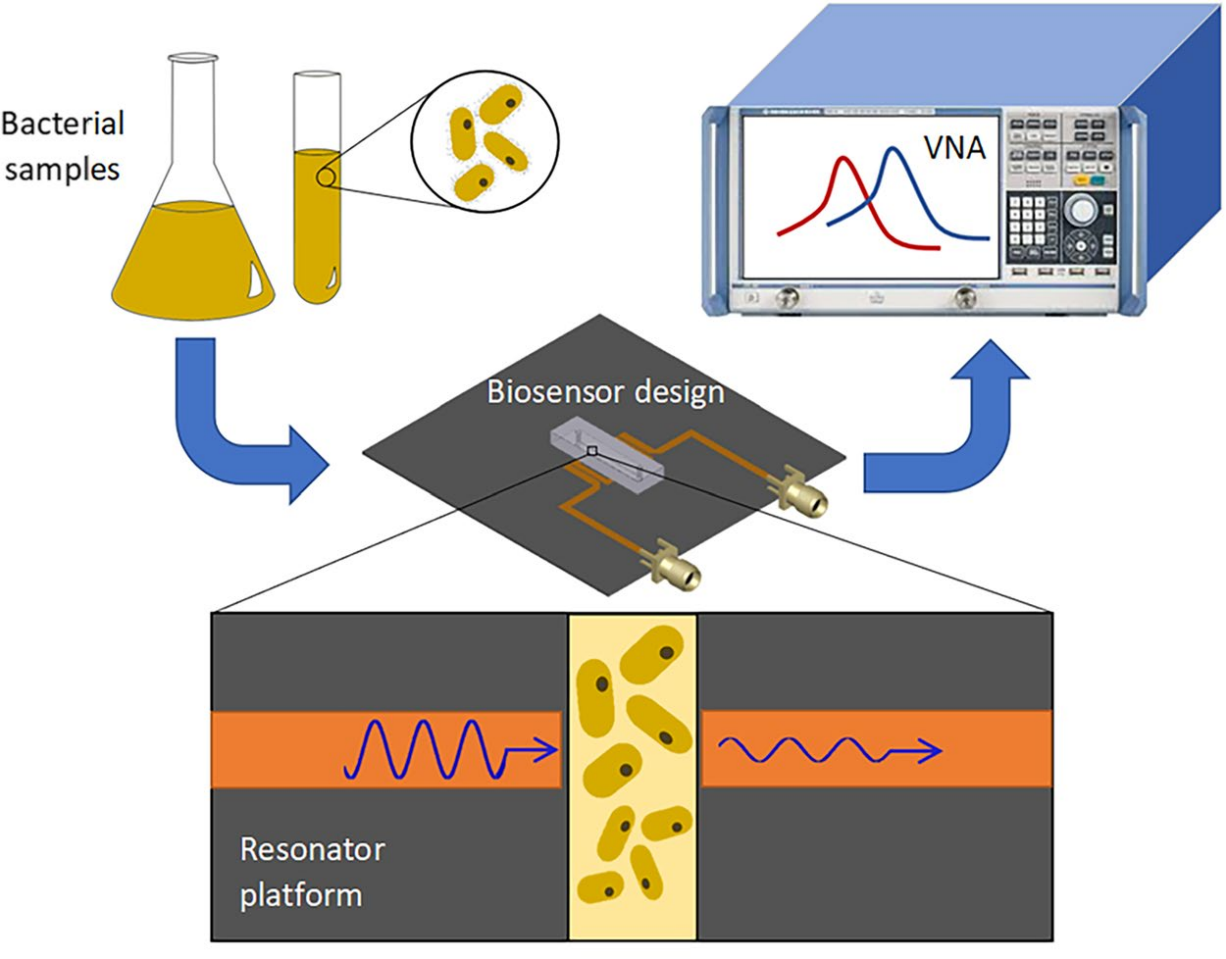
Conceptual representation of the experiments for the detection of bacteria concentration and proliferation. The cultured bacteria are diluted to a desired concentration and introduced into the microwave-microfluidic platform. The electrical signal of the resonator is analyzed through a vector network analyzer (VNA) to gather resonant profile for bacteria in different concentrations and environmental pH, and for long-term screening of their growth.

In this study, a simplified model of bacteria concentration and growth analysis within various environments is presented. The *Escharichia coli* (*E. coli*) MG1655 bacteria with the OD_600_ concentration ranging from 0.003 to 1 are injected into the microfluidic chip resting on top of the resonator (Fig. 1). The electrical signal of the resonator is then analyzed by means of a vector network analyzer (VNA). The advantages of this work include real-time, non-contact, accurate and reliable testing of bacteria concentration in a variety of environments and bioassays. It also has the potential to be improved to perform real-time AST in a point-of-care setting. The bacteria concentrations tested in this work are equivalent to clinical concentration of bacteria presently handled in clinical microbiology laboratories, which is typically greater than 10^5^ CFU/mL, although clinics can require high volumes of patient samples^42,43^. Given the possible presence of bacteria in a plethora of environments within the human body^44^, the effect of environmental factors need to be considered for developing biosensors for the diagnosis of bacterial infections. pH value is a strong indicator of the environmental conditions, which greatly affects metabolic activities of bacteria and therefore the ability to proliferate and fatality potential to patients^45^. The bacteria in pH environments of 5.5–8 are examined in increments of 0.5 to calibrate the biosensor. This pH range fits well within the pH of human biofluids, although most biofluids are around the generally accepted physiological pH of 7.4. Furthermore, monitoring the growth of bacteria is studied as a function of time to assess the sensor’s performance in detecting the bacteria proliferation under transient conditions. This study allows us to further develop a rapid, label-free and contactless diagnostic tool for clinical analysis of biofluids in clinical microbiology laboratories for both rapid detection of bacteria and screening the interaction of bacteria and antibiotics.

## Results and Discussion

### Electromagnetic Field Analysis of the Sensor

**Figure 2:**
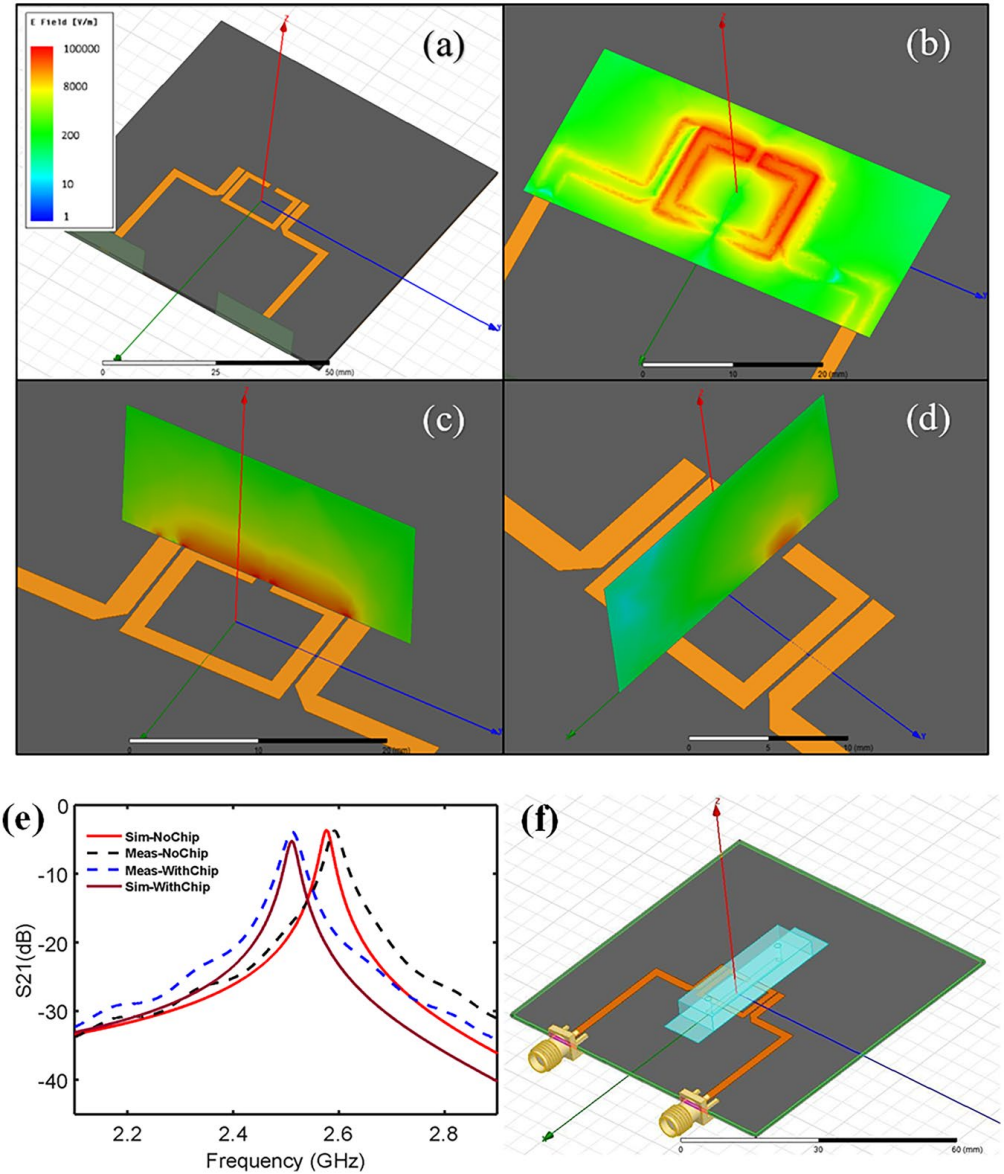
High frequency structure simulator (HFSS) electrical field simulation results of the bare resonator. (**a)** The bare geometry of the sensor, (**b)** the electrical field simulation at the surface of the resonator parallel to the sensor plane, (**c)** the electrical field simulation perpendicular to the sensor plane and along the ring structure, (**d)** the electrical field simulation perpendicular to the sensor plane and along the sensitive region of the sensor, (**e)** HFSS simulation results compared with experimental S21 parameters gathered from testing the resonator alone and with the microfluidic chip assembled. (**f**) HFSS depiction of the sensor geometry and its entirety.

A ring resonator structure was implemented in High Frequency Structure Simulator (HFSS) software to conduct analysis on the electrical fields surrounding the microwave platform. This 2-port structure was designed to be operated under 2–3 GHz frequency span with an initial quality factor of 83.58. Excitation through one port and receiving power from the other port was used to analyze the absorption of microwave power within the sensor’s vicinity. Figure 2a displays the simulated structure within the HFSS software. According to the simulation results, the electric field is highly concentrated around the microstrip resonator gap within the ring structure. This gap area, particularly on the surface of the resonator, is the most sensitive region to the variation within the sensor’s environment (Fig. 2b). Electrical field analysis was conducted in two planes perpendicular to the surface of the resonator to demonstrate the penetration distance of the electrical field for the sensor at the resonant frequency (Fig. 2c,d). It is, therefore, implied that the sensitive region of the resonator has the capability to handle a volume of 0.38 µL.

The measured profiles of S21 parameters are compared with the simulated results through HFSS of two scenarios: the bare resonator, and the resonator assembled with the microfluidic chip (Fig. 2e). A close relationship between the resonant profile of the simulation and experimental data is demonstrated (Fig. 2d). The resulting resonant frequency and amplitude of the simulation was found to be 2.576 GHz and −3.658 dB, respectively, while the experimental data for the resonant frequency and amplitude are determined to be 2.591 GHz and −3.698 dB, respectively, resulting in a 0.58% and 1.09% error between the experimental and theoretical values. Following the validation of the numerical model, the resonator integrated with the microfluidic chip was simulated to have a resonant profile of 2.511 GHz and −5.25 dB (Fig. 2f). The experimental data was then gathered to be 2.511 GHz and −3.882 dB, showing a 0% and 26.05% error of the resonant frequency and amplitude, respectively. These errors, although within reasonable bounds, can be caused through differences between the simulated and real permittivity, loss factors of the thin PDMS layer and ultra-thin glass, and deviations of height of the bulk PDMS structure. Furthermore, spatial differences between the experimental setup and theoretical simulations have a considerable effect on these values, therefore the exact placement of the microchannel above the resonator will also play a role in the small numerical and experimental errors. These errors, however, do not affect the subsequent results as they are proposed as baseline results for the following tests with the resonator in the presence of fluids and bacteria.

### Experimental Setup of the Sensor and VNA

**Figure 3:**
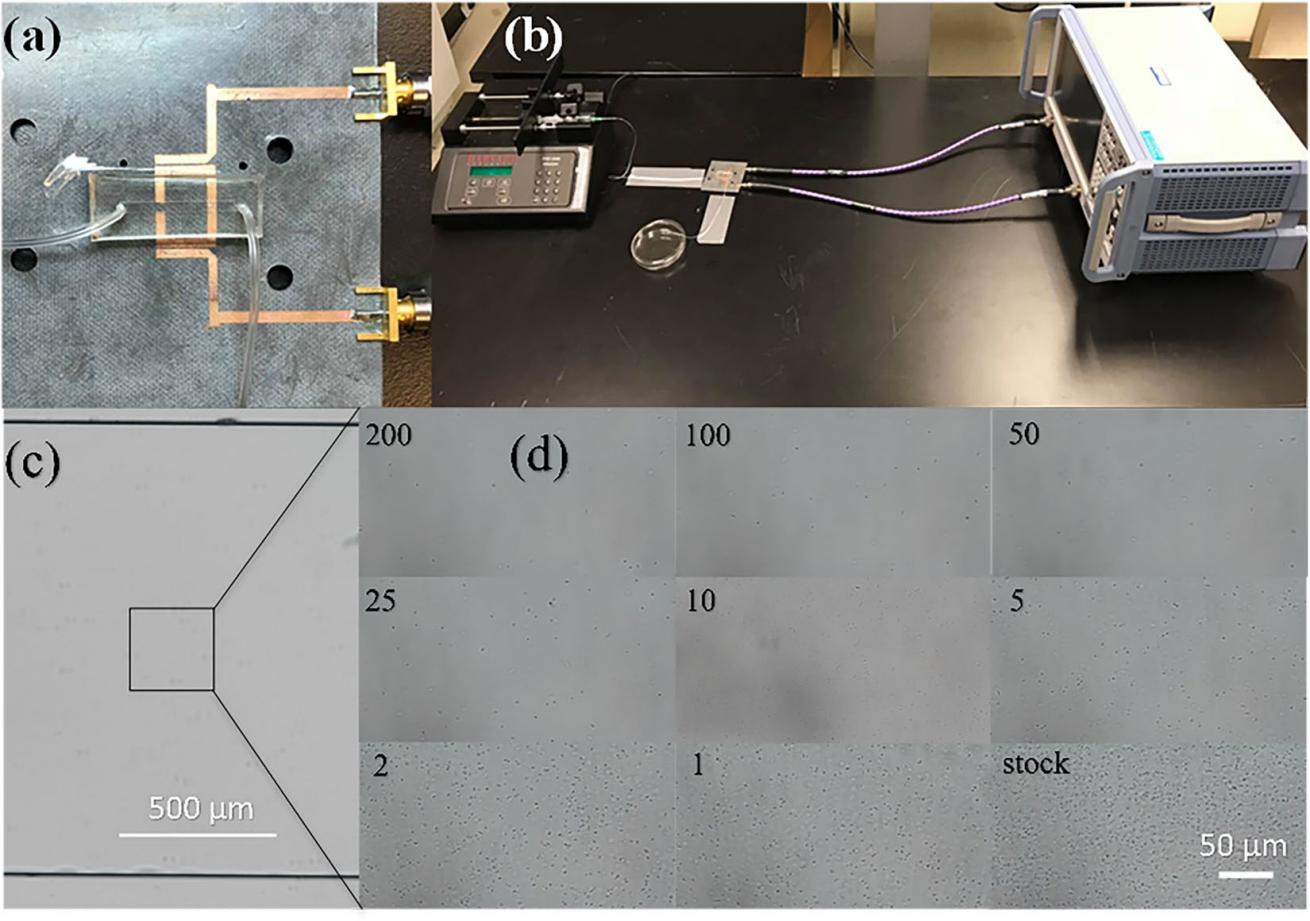
The sensor, experimental setup, and bacteria imaged within the microfluidic chip. (**a**) The closeup view of the microwave resonator with the microfluidic chip resting on top secured by double sided duct tape. (**b)** The experimental setup that includes the syringe pump, flow tubing, microwave setup, waste collection and vector network analyzer (VNA) system. (**c**,**d**) Visual illustration of *E. coli* bacteria at an initial OD_600_ value of 1.6 and pH 7.5 on top of the active region of the sensor. (**c**) The bare microfluidic channel while, (**d**) the subsequent figures show each dilution factor as labelled inside the chip.

The room temperature was set to 20 °C for all sets of experiments, unless otherwise mentioned. The cables and tubing were secured through duct tape to limit the movement and mechanical drift of data. The microfluidic chip was secured onto the resonator using double-sided tape (Fig. 3a). The entire setup can be seen in Fig. 3b. Temperature variations would require the sensor to be calibrated for the testing temperature. The VNA was brought to operating temperature and calibrated within the frequency span of 2–3 GHz at this temperature using 2001 steps in transmission mode, with an IF bandwidth of 1 kHz. The resonant frequency and amplitude were extracted through S21 parameters for different bacteria concentrations at distinct pH levels. The heat generated at these settings was miniscule and can be neglected at any portion of the analysis. The response from the VNA was nearly immediate but measurements were taken at 1-minute intervals to ensure homogenous distribution of bacterial fluid within the chip.

### Microwave and Imaging-based Detection of Bacterial Concentration

For detecting bacteria concentrations using microscopy technique, various concentrations of *E. Coli* were prepared (in pH 7.5 and the initial OD_600_ value of 1.2) and injected into the microfluidic chip at dilution factors of 0, 1, 2, 5, 10, 25, 50, 100 and 200. When the fluidic channel is filled with the liquid, the flow is stopped and the bacteria within the channel were imaged immediately by the Nikon AR1 microscope. Figure 3c shows a visual inside the bare microfluidic channel. The concentrations of bacteria present above the active region of the sensor increases with decreasing dilution factors in subsequent steps of the experiment (Fig. 3d). The concentration of bacteria detected within the microchannel for different dilution factors is determined to be proportional to the concentration of bacteria injected into the chip, confirming the uniform distribution of bacteria within the microfluidic chip. It is noted that no bacteria were found adhered to PDMS surfaces and this is important to maintain the homogenous representation of each dilution factor. The flowrate of 50 µL/min through the channel was high enough to produce enough sheer to prevent cells from sticking to the channel walls. This was observed through by fixating the camera view on the channel wall and through multiple iterations of replacing fluid with higher bacterial concentrations, no cells were found to adhere to the channel walls.

**Figure 4:**
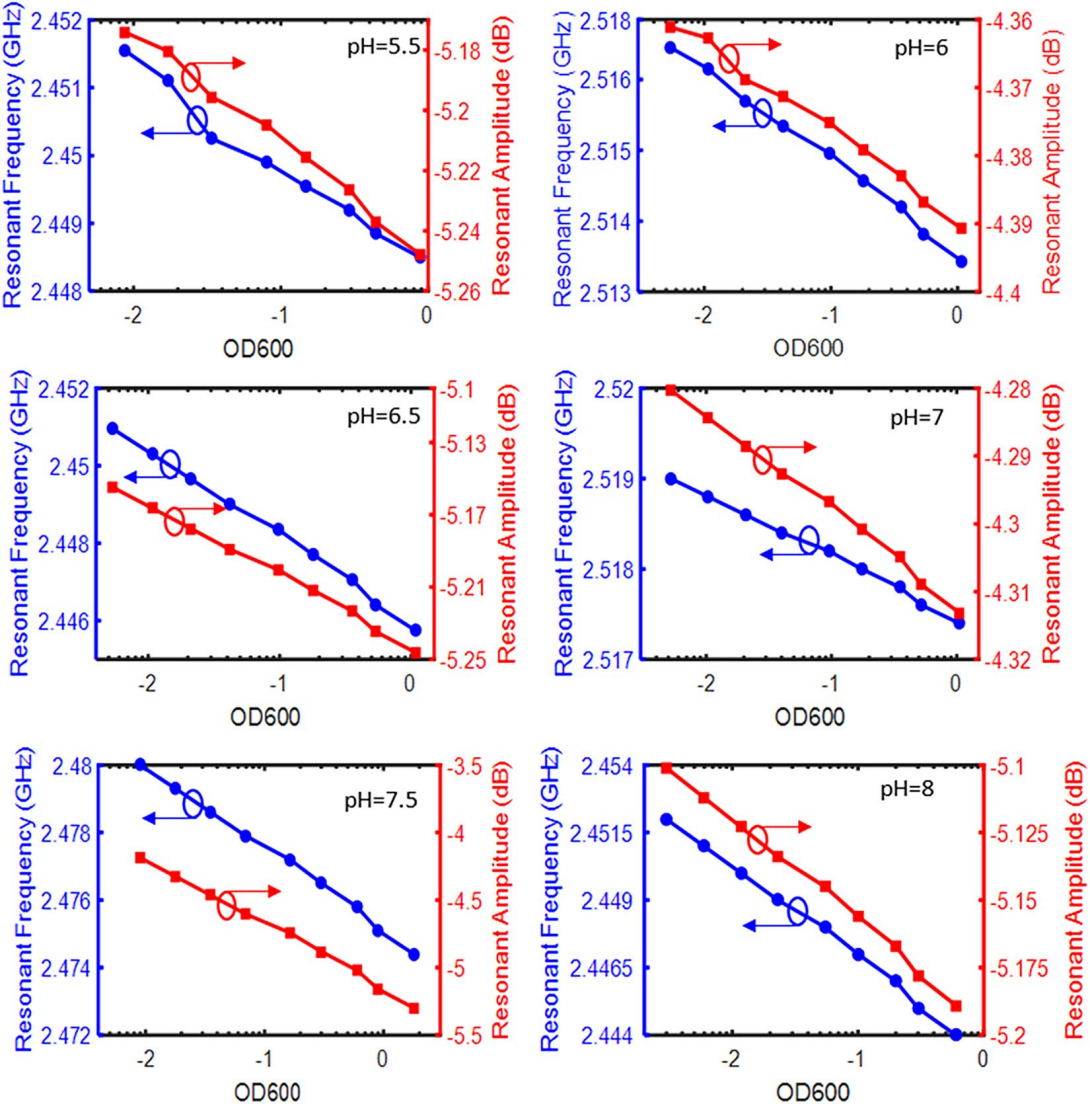
Resonant amplitude and frequency for different OD_600_ values recorded by the VNA for different pH values of 5.5, 6, 6.5, 7, 7.5, 8. The sensor response is highly linear for a wide range of bacteria concentrations and environmental pH values. The error bars are too small to be seen.

As shown in Fig. 4, a highly linear relationship of resonant amplitude and resonant frequency is demonstrated with respect to the logarithm of OD_600_ values of the solution with pH 7.5 residing inside the microfluidic chamber above the resonator’s active region. The linear response of the resonator to the logarithm of OD_600_ values is also extended to all other pH values (Fig. 4). We examined the resonator response to pH values within the range of 5.5–8 with increments of 0.5 and OD_600_ values ranging from 0.003 to approximately 1 (Fig. 4). Each test was measured three times and the error bars are displayed (the error bars are too small to be seen in Fig. 4). The trends clearly display a decreasing resonant frequency and resonant amplitude with the increase of OD_600_ values. All relationships displayed R^2^ values of greater than 0.99 except for the resonant frequency plot of pH 5.5, which displayed an R^2^ value of 0.9832. This response is highly linear and reliable; however, a higher variance was detected in resonant frequency response due to introducing excess protons, metabolites and proteins of these cells. Moreover, detection of changes in resonant frequency and resonant amplitude is registered immediately, therefore providing a strong avenue towards rapid AST and diagnosis. It is noted that the difference in resonant frequency and resonant amplitude between each pH can be explained by the miniscule spatial differences in placing the microchannel on the resonator and the size variations in PDMS blocks.

The slope of each line is an indicative of the sensitivity of the device (the resonant frequency and resonant amplitude) to OD_600_, and hence the bacterial concentration. It is expected that each solution interacts with the electrical field differently as each pH inherently differs in charge. This may have effects on the sensitivity of the resonator, as impedance and permittivity both effect the electrical field interactions. Subsequently, the resonator can be further optimized with this knowledge to test under different environments by utilizing a higher frequency range or different geometry. The graph with pH 8 shows the highest slope magnitude of −0.0034 GHz/log(OD_600_) in resonant frequency displaying a large sensitivity to bacterial concentrations. Meanwhile, the graph with pH 7 shows a slope of −0.0007 GHz/log(OD_600_), which is less sensitive to bacterial concentration. Similarly, the graph with pH 7.5 has a large sensitivity to bacterial concentrations with respect to resonant amplitude (−0.4771 dB/log(OD_600_)), and the graph with pH 6 has the lowest sensitivity with respect to resonant amplitude (−0.0130 dB /log(OD_600_)).

### Microwave-based Screening of Bacterial Growth

**Figure 5:**
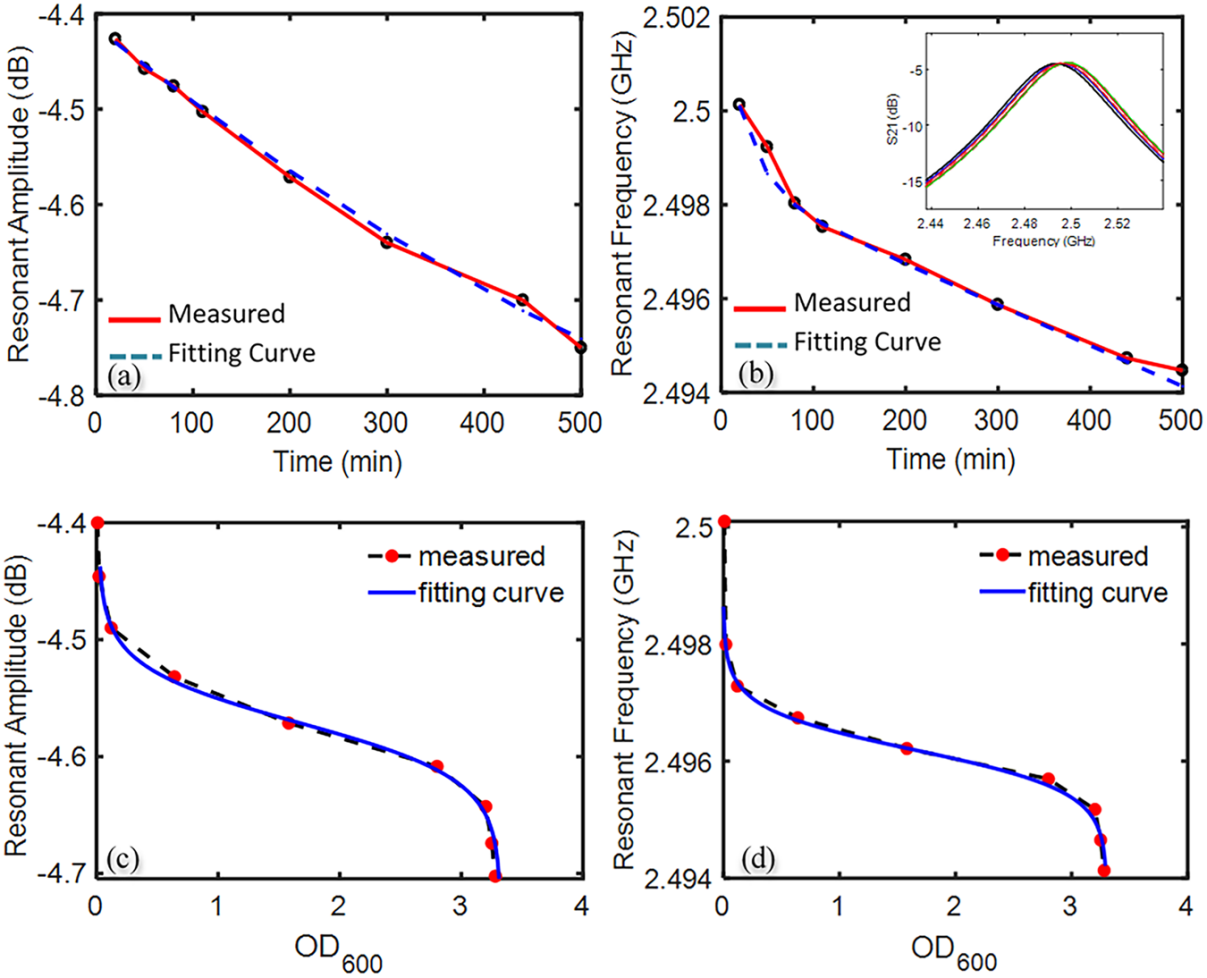
The response of the resonant bacteria profile as a function of time. (**a**) The change in the resonant amplitude versus time over the span of 500 min, (**b**) the change of resonant frequency versus time over the span of 500 min, (**c)** the change of resonant amplitude as a function of OD_600_, which was calculated through conducting a transient experiment of bacteria growth (OD_600_ change) and comparing the fitted curve with resonant data, (**d**) the change of resonant frequency versus the OD_600_ which was calculated in a similar respect to (**c**).

A similar setup used for the microwave-based detection of bacterial concentration (Fig. 3) was used to screen the bacterial growth, with the sole difference being the sensor was placed inside a New Brunswick Scientific Innova 40 shaker incubator, to maintain the atmospheric temperature at 37.4 °C. This temperature is the recommended culturing temperature at which the proliferation of *E. Coli* is the fastest. Bacteria injected into the chip of pH 7.5 and OD_600_ 0.008 were left inside the microfluidic channel and incubated for several hours. This pH was selected, as pH 7.5 is the closest to human body’s physiological pH of 7.4. Furthermore, it was also the second most sensitive with respect to resonant frequency, after pH 8. The tubing was crimped at both the inlet and outlet, and data was gathered every 10 min over a 500-minute span. The results show that both the amplitude and frequency decrease with time, consistent with the observed trends in experiments previously conducted in this study. The resulting data of bacteria growth using the resonator was fitted with exponential curves resulting in R^2^ values of 0.997 and 0.9893 for resonant amplitude and resonant frequency, respectively. The resulting equations (1) and (2) for each of the graphs (Fig. 5**)** is indicative of the lag-log-stationary growth model of bacterial cells. However, several variables other than the bacteria growth may contribute to the signals, e.g. the metabolic reactions, pH and CO_2_ change, nutrient concentration in the MH medium, and aggregation and colonization of the bacteria during the bacteria culture. These variations in the sensor’s response can be initiated by the permittivity and loss factor inside the microfluidic channel. It is demanding to distinguish the response of each of these individual factors through simple S parameter analysis in a complex biological environment. However, they can be utilized as an indication for overall metabolic activity of the bacterial sample. While the results of Fig. 5 are demonstrated to be highly predictive and potentially applicable for clinical use, multiplexing of the microwave resonator and microchannel is one solution for in-depth analysis of these factors. Combining multiple ring resonators in a microwave design allows one section to act as a control to be compared to another^46^, which could lead to deeper levels of analysis on specific factors.

Further experiments were done to culture bacteria over an 8-hour period. OD_600_ values were recorded every hour, starting from 0.008, and the same lag-log-stationary growth model was shown to be prevalent (shown in Supplementary Figure S1). It was found that upon comparing the resonant data with the OD_600_, the extreme ends of the concentration of bacteria tended to deviate from linearity, displayed in Fig. 5c,d. This is shown by comparing the transient OD data with the corresponding resonant data. The slower growth rate at the start and end of the experiment is not as prevalent in the resonant data as slope of the resonant frequency does not deviate proportionally to the transient OD changes and the slope of the resonant amplitude barely shifts at all. However, during the middle of the experiment, as OD reaches ~0.05, the resonant data compared to the OD displays a near linear relationship until the OD reaches ~3.2, where linearity of data is once again compromised. Further optimization of the sensor is required to increase the sensitivity and widen the linear detection range.

1 $$\begin{array}{cc}{\rm{f}}\,({\rm{t}})=20.3\times {{\rm{e}}}^{(0.0005933\cdot {\rm{t}})}-24.73\times {{\rm{e}}}^{(0.0005192\cdot {\rm{t}})} & ({\rm{Resonant}}\,{\rm{amplitude}})\end{array}$$

2 $$\begin{array}{cc}{\rm{g}}({\rm{t}})=0.001797\times {{\rm{e}}}^{(-0.03534\bullet {\rm{t}})}+2.4983\times {{\rm{e}}}^{(-3.477{\rm{e}}-06\bullet {\rm{t}})} & ({\rm{Resonant}}\,{\rm{frequency}})\end{array}$$

It is noted that the concentration initially cultured in this work is far below the technical capacity of clinics which demonstrates the potential of this device in a clinical setting. Seeing as clinical samples require approximately 48 hours to culture, utilizing this technology to display change in culture samples can prove to be beneficial. Having deemed that the overall metabolic activity of solutions changes with the proliferation in bacteria, utilizing the VNA can help indicate the presence of bacteria in a matter of minutes, as opposed to standard clinical practices. Furthermore, the value of non-invasive and contactless features are assets, as this increases sterility in a hospital setting. Our contactless bacteria sensing device could further be adapted to be used directly on patient samples.

In summary, a microfluidic-integrated microwave biosensor for detecting the concentration and proliferation of *E. Coli* in real-time was presented and tested. Our sensing platform possesses several key advantages such as low sample and reagent volume requirements (~400 nL), enhanced detection, sensitivity (detecting OD600 values of 0.003, without confirming the limit of detection), rapid detection times (almost immediately), and enhanced combinatorial capabilities compared to several alternative electrical sensing techniques. Moreover, our method further enables direct observation and enumeration of bacteria. Images of the bacteria suspended within the microchannel show a uniform distribution of bacteria all along the channel and without any adherence to the PDMS surface. This work gives us an insight to the dielectric properties of *E. Coli* K12 MG1655 and how they can be correlated to the resonant amplitude and resonant frequency of the resonating sensor.

Given the highly linear relationships between the electrical signal and the concentration of bacteria, further tests need to be established using our microwave-microfluidic platforms for developing diagnostic methods and antibiotic susceptibility testing (AST). This work could benefit clinical microbiology laboratories through automating the workflow of AST and increasing their capabilities for the diagnosis and handling bacterial infections. Future work will focus on improving the layout of the electrodes and reducing the distance between the microwave resonator and the microfluidic channel to detect *E. Coli* at lower concentrations. Further experiments need to be performed to assess the feasibility of rapid diagnosis and management of infections. Moving beyond our present work, the ability of the microfluidic-microwave platform to support multiplexed and high-throughput cell culture and sensing over extended periods of time (up to several hours) in a non-invasive and noncontact system makes it a versatile tool for time resolved sensing of live cells in a multiplexed fashion for other applications, including cancer detection and systems biology.

## Methods and Materials

### Reagents and Materials

Sodium phosphate dibasic (795410) and sodium phosphate monobasic (RDD007) for pH adjustment were purchased from Sigma Aldrich, Canada. Mueller Hinton Broth BBL (211443) as growth media (GM) was purchased from BD, Canada. Ammonium persulfate was purchased from MG Chemicals (Surrey, Canada). The polydimethylsiloxane (PDMS) and curing agent were obtained from Dow Corning Slygard. Ultra-thin 70 µm thickness AF32 glass was kindly provided by Schott Glass USA. Tygon Microbore tubing, 0.020″ × 0.060″OD, 100 ft/roll was purchased from Cole-Parmer, Montreal, Canada, PCB Board was provided by Rogers Corp. USA. Also, a Rohde and Schwarz ZNB20 VNA was used for the analysis of the resonator signals.

### Bacterial Samples Preparation

The bacteria strain utilized in this work is wild-type strain DA5438 (*E. Coli* MG1655). In preparation for analysis, the *E. Coli* from 50% glycerol stocks at −80 °C were inoculated into 50 mL Müller-Hinton (MH) growth medium and incubated (37 °C; shaking at 170 RPM) for about 10 hrs. Subsequently, the optical density (OD_600_) was measured using NanoDrop™ One Microvolume UV-Vis Spectrophotometer from Thermo Fisher Scientific. The pH was measured for each sample by an Orion™ Versa Star Pro™ pH Benchtop Meter (Thermo Fisher Scientific). A mixture of 0.2 M sodium phosphate dibasic and 0.2 M sodium phosphate monobasic was prepared for pH adjustment of bacterial samples and created solutions with pH 5.5, 6, 6.5, 7, 7.5, 8. The MH medium of the same pH was used to further dilute the bacteria samples by factors of 1, 2, 5, 10, 25, 100 and 200, since these dilution factors would bring the concentration of bacteria down to the capacity that cannot be handled by clinics. It is noted that the bacteria were stored in 4 °C while they were not in use to retard their growth to ensure the most accurate representation of each dilution factor. The samples were brought to room temperature prior the use through dilution in MH medium. For high concentrations of bacteria, 2–3 mL of MH medium was left at room temperature for about 3 min to register room temperature.

### Microfluidic Platform

The microfluidic platform primarily consisted of PDMS and glass as these are robust materials in microfluidic research with stable electrical properties, when subjugated to electromagnetic fields. The microfluidic chip features a simple straight channel produced from 10:1 ration of polydimethylsiloxane (PDMS) to curing agent. The channel was 2 mm wide, 0.17 mm high and 23 mm long, and capable of handling 7.82 µL of fluid. This chip was produced from a silicon wafer mold with the SU8 straight channel features hard-baked onto the substrate through photolithography^45^. The PDMS polymer was poured over the mold and baked at 80 °C for 30 min. The chip was carved out and 1.5 mm holes were punched into the hardened polymer. The chip was subsequently baked for 24 hrs. Simultaneously, the PDMS polymer of the same polymeric ration was spin-coated over ultra-thin glass (70 µm in thickness), after being cleaned with acetone and nitrogen, to form an additional layer of 25 µm. Ultra-thin glass was used due to its mechanical rigidity, maintaining the shape and size of the PDMS-based chips over long experiments after the bonding had occurred, given the fact that the microwave sensing technology is very sensitive to spatial variations. The 70 µm thin glass layer was used to bring the fluid inside the chip as close as possible to the resonator, to allow for greater accuracy in measurement, while maintaining the robustness of the design, as PDMS covered thin-glass would not shatter easily and the microfluidic chip would bond to the PDMS surface very easily, reducing the likelihood of leak through the channel. The thin PDMS layer was cured in oven for a 24 hr period. Following the curing of both the fluidic network layer and the thin PDMS layer over the glass slide, the bonding surfaces were thoroughly cleaned with acetone and nitrogen. The featured side of the chip was then bonded irreversibly with the thin PDMS layer on the glass through oxygen plasma treatment by Electro-Technic Products Inc. BD 20 Plasma wand to create a hydrophilic surface. Tygon tubing, 1.5 mm outer diameter, was connected to the holes to bring flow into and out of the chip. Syringes with fluids were mounted on Harvard Apparatus syringe pumps and connected to the tubing. The waste was collected in a petri dish and discarded appropriately. The resulting fluidic device was then mounted onto the resonator through double sided tape placed away from the resonator’s sensitive zone.

### Microwave Resonator

A microstrip planar ring resonator sensor was implemented on a high frequency RO5880 substrate from Rogers^26^. The substrate was covered by two copper layers on its top and bottom surface with the thickness and conductivity of 35 µm and 58 MSm^−1^, respectively. The substrate had a thickness of 0.79 mm with the permittivity of 2.2 +/− 0.02 and loss tangent of 0.0009.

### Microwave Measurements of Varying pH and Concentrations

The electrical measurements of the bare resonator and the resonator placed underneath the microfluidic channel were taken before the start of the experiments with bacteria. Syringes were filled with MH medium and connected to the microfluidic chip using Tygon tubing. The fluid was introduced into the microchannel while the VNA was running. The syringes were set onto the syringe pump calibrated to the flow rate of 50 µL/min and run for two minutes. A flowrate applied was to ensure a reduced attachment of bacteria to PDMS surfaces. Every following concentration was therefore ensured to be a homogenous representation of its dilution factor. Subsequently, three measurements were then recorded from the VNA with one-minute time intervals. The syringes were then replaced with a higher concentration of bacteria; as the fluid as once again forced in, flushing out all the fluid of the previous concentration, and set onto the syringe pump at the same flow rate. This set of experiments was repeated for all pH values of the medium and bacteria concentrations. The plasma treated hydrophilic surface of the PDMS helped eliminate air bubbles as subsequent fluids were forced in.

### Screening of Bacteria Growth

The resonator was placed inside a New Brunswick Scientific Innova 40 shaking incubator at the temperature of 37.4 °C, and the electrical measurements of both the bare resonator and resonator with the microfluidic chip were taken. Subsequently, bacterial cultures of OD_600_ of 0.008 was injected into the microfluidic chip using a similar setup to that described above. However, this experiment was done without flow, as the two ends of tubing were crimped to contain all the bacterial sample inside to proliferate. The electrical measurements of these samples were obtained in 10-minute intervals for 500 min, as this is sufficient time for *E. coli* to proliferate into the log phase of growth. This sequencing frame was chosen as the doubling time of *E. coli* MG1655 in MH medium is approximately 20 min^5^. Therefore, the selected 500 min experimental period provides a reasonable growth curve in terms of resonant frequency and amplitude.

## Electronic supplementary material

Dataset 1
