# Supplementary material for: Sensitive, Real-time and Non-Intrusive Detection of Concentration and Growth of Pathogenic Bacteria using Microfluidic-Microwave Ring Resonator Biosensor
Source: Sci Rep. 2018 Oct 25;8:15807. doi: 10.1038/s41598-018-34001-w (PMC6202403; doi:10.1038/s41598-018-34001-w)
Supplement: Supplementary file 1 — Dataset 1 [file 41598_2018_34001_MOESM1_ESM.docx]

**Supplementary Information**

**Sensitive, Real-time and Non-Intrusive Detection of Concentration and Growth of Pathogenic Bacteria using Microfluidic-Microwave Ring Resonator Biosensor**

Rakesh Narang^1,3,4^, Sevda Mohammadi^2+^, Mehdi Mohammadi Ashani^1,3,4+^, Hamid Sadabadi^1,5^, Hossein Hejazi^6^, Mohammad Hossein Zarifi^2*^, Amir Sanati-Nezhad^1,3,4*^

*1 BioMEMS and Bioinspired Microfluidic Laboratory, Department of Mechanical and Manufacturing Engineering, University of Calgary, Calgary, AB T2N 2N1, Canada*

*2 Microelectronics and Advanced Sensors Laboratory, School of Engineering, University of British Columbia, Kelowna, BC V1V 1V7, Canada*

*3 Biomedical Engineering Graduate Program, University of Calgary, 2500 University Dr. NW, Calgary, AB T2N 1N4, Canada*

*4 Center for BioEngineering Research and Education, University of Calgary, Calgary, AB T2N 1N4, Canada*

*5 Wireless Fluidics Inc, Edmonton, AB Canada*

*6 Subsurface Fluidics and Porous Media Laboratory, Chemical and Petroleum Engineering, University of Calgary, Calgary, AB T2N 1N4, Canada*

Corresponding Authors:

Amir Sanati-Nezhad, Department of Mechanical and Manufacturing Engineering, BioMEMS and Biopinspired Microfluidic Laboratory, University of Calgary, EEEL 455B 2500 University Drive NW, Calgary, Alberta T2N 1N4, Canada. Email: [amir.sanatinezhad@ucalgary.ca](mailto:amir.sanatinezhad@ucalgary.ca)

Mohammad Hossein Zarifi, School of Engineering, The University of British Columbia, Kelowna, BC, V1V 1V7, Canada, Email: [mohammad.zarifi@ubc.ca](mailto:mohammad.zarifi@ubc.ca)

^+^ Equal contributions

**Figure S1:** Graph displaying bacteria growth measured through optical density (OD_600_) over 8 hours.
